# Psychophysiological Correlates of Sexually and Non-Sexually Motivated Attention to Film Clips in a Workload Task

**DOI:** 10.1371/journal.pone.0029530

**Published:** 2011-12-21

**Authors:** Sandra Carvalho, Jorge Leite, Santiago Galdo-Álvarez, Óscar F. Gonçalves

**Affiliations:** 1 Neuropsychophysiology Lab, Center for Research in Psychology (Cipsi), School of Psychology (EPsi), University of Minho, Braga, Portugal; 2 Department of Clinical Psychology and Psychobiology, University of Santiago de Compostela, Santiago de Compostela, Spain; University of Sydney, Australia

## Abstract

Some authors have speculated that the cognitive component (P3) of the Event-Related Potential (ERP) can function as a psychophysiological measure of sexual interest. The aim of this study was to determine if the P3 ERP component in a workload task can be used as a specific and objective measure of sexual motivation by comparing the neurophysiologic response to stimuli of motivational relevance with different levels of valence and arousal. A total of 30 healthy volunteers watched different films clips with erotic, horror, social-positive and social-negative content, while answering an auditory oddball paradigm. Erotic film clips resulted in larger interference when compared to both the social-positive and auditory alone conditions. Horror film clips resulted in the highest levels of interference with smaller P3 amplitudes than erotic and also than social-positive, social-negative and auditory alone condition. No gender differences were found. Both horror and erotic film clips significantly decreased heart rate (HR) when compared to both social-positive and social-negative films. The erotic film clips significantly increased the skin conductance level (SCL) compared to the social-negative films. The horror film clips significantly increased the SCL compared to both social-positive and social-negative films. Both the highly arousing erotic and non-erotic (horror) movies produced the largest decrease in the P3 amplitude, a decrease in the HR and an increase in the SCL. These data support the notion that this workload task is very sensitive to the attentional resources allocated to the film clip, although they do not act as a specific index of sexual interest. Therefore, the use of this methodology seems to be of questionable utility as a specific measure of sexual interest or as an objective measure of the severity of Hypoactive Sexual Desire Disorder.

## Introduction

Sexual motivation is an internal state influenced by a complex relationship among several factors that determine engagement in sexual activity [1], [2], [3], [4]. To understand the psychophysiological correlates of sexual motivation and behavior, the effect of exposure to visual sexual content on both central nervous system (e.g., Event Related Potentials - ERP) and peripheral measures (e.g., Skin Conductance Level ) has been widely studied [5], [6].

Of the central nervous system variables, the P3 ERP component has been the focus of much attention [7]. The P3 is a large positive potential that increases in amplitude from the frontal to parietal electrode sites [8], [9] and is believed to be an index of neural activity involved in updating stimulus representations [10]. The P3 amplitude usually decreases by increasing the cognitive demands of the task as well as with the presentation of distractor stimuli [11], [12]. Several studies have shown that this component is enhanced during visual exposure to both pleasant and unpleasant stimuli [13], [14], [15]. These results have been interpreted as evidence that both attention and working memory are modulated by motivationally relevant stimuli [16], [17].

There is also evidence for the effects of motivationally relevant stimuli at the peripheral level [18]. Several studies have reported that specific peripheral responses (e.g., galvanic skin response, GSR, and heart rate, HR) are sensitive to either the valence or arousal dimensions of the stimulus [19], [20], [21]. Although some studies [18] highlight that psychophysiological responses can be modulated as a function of the emotional content of the stimulus, both the defensive and appetitive systems can be activated by stimulus of high motivational relevance compared to those that have less motivational relevance [22]. Some authors, however, claim that the P3 component is particularly sensitive to pictures of sexual content and is, therefore, a potentially specific psychophysiological signature of sexual interest and desire [23], [24]. For instance, the P3 amplitude was found to be largest during passive erotic picture viewing when contrasted with the viewing of pictures containing sports content [7]. There is also evidence of an increase in the amplitude of several other ERP components in females during exposure to erotic visual stimuli when compared to neutral, negative and other positive visual stimuli, starting as early as 195 ms (Cz) post-onset of the stimulus and up to 1800 ms after viewing of the image [25].

These results have led some authors to explore the potential use of the P3 component, using a workload task, as an objective measure of sexual interest and as a diagnostic tool for sexual desire [23], [24].

In a recent study by Vardi and colleagues [23], 30 healthy participants (males and females) performed a binaural auditory oddball task while watching film clips of three different types (i.e., scenery, sport and sexual content). The largest decrease in the P3 amplitude occurred while participants watched film clips with sexual content. These results led the authors to suggest that the P3 could be used as an objective measure to quantify the cognitive dimension of sexual arousal. At a later point [24], the authors tested the utility of the P3 as diagnostic assessment of sexual interest. Thirty sexually healthy females and twenty-two females with sexuality disorders (FSD) were compared using a similar workload design (i.e., an auditory oddball paradigm where participants were watching sexual film clips with or without sexual intercourse content). Whereas healthy controls demonstrated similar P3 amplitude reduction for both sexual category films (intercourse and non-intercourse), the FSD patients had a greater P3 amplitude reduction while watching films without intercourse content. This result was interpreted as the specific emotional impact of that particular content on the FSD patients. That is, the hedonic valence of non-intercourse film clips would be a higher predictor of motivated attention than the hedonic valence of sexual intercourse film clips given the specific non-interest in terms of intercourse in women with FSD.

From these studies, the authors concluded that the P3 component could be used as an objective measure of sexual interest, constituting a promising diagnostic tool for clinical practice. However, despite these promising results, we believe that some methodological limitations make the validity of these conclusions questionable. Particularly important is the fact that neither valence nor arousal levels of the stimuli were controlled. In fact, previous research has shown that different levels of arousal and valence constitute important modulators of the P3 component [26].

The aims of this study are three-fold. The first is to assess the modulation of the P3 component in a workload task as a primary outcome measure by comparing changes in the P3 amplitude between sexual and non-sexual film clips with different valences and levels of arousal. The second is to use the heart rate (HR) and skin conductance level (SCL) as secondary outcome measures, as both measures reveal the peripheral reactivity of the participants to the sexual and non-sexual film clips and may increase our understanding of the primary outcome measure. Finally, the third aim is to test the utility of this methodology as a potential index of sexual motivation.

## Results

### The Behavioral Responses

In terms of behavioral responses as measured by the percentage of correct responses to the infrequent stimuli of the oddball the mixed model ANOVAs showed a significant difference in the percentage of correct responses (F(4,112) = 8.139, ε = .294, p = .005). The multiple pairwise comparisons showed that a higher percentage of errors took place during horror film clips (M = 98.378%, SE = .511) compared to both the erotic film (M = 99.910%, SE = .090) (p = .042) and the social-negative clips (M = 100%, SE = .000) (p = .036). No gender differences were found (F(1,28) = .962, p = .335).

The mixed model ANOVAs showed a significant difference in the Reaction Times between categories (F(4,112) = 6.476, ε = .583, p = .002). The multiple pairwise comparisons showed that RTs were longer for the targets while observing horror film clips (M = 351.435, SE = 10.782) when compared with auditory alone stimuli (M = 328.930, SE = 7.966) (p = .021). No gender differences were found (F(1,28) = .365, p = .550).

### The Neurophysiological Responses

This section presents the results for P3 in the Cz electrode site and the regional analysis, in the 300–400 msec time window, elicited by an auditory oddball task, while participants viewed film clips (please see the Methods section for further details).

#### Effects of film clip categories×Gender on P3 amplitude at Cz

**Figure 1:**
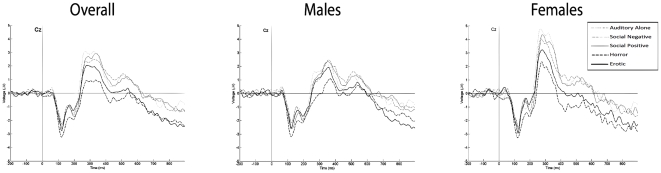
Averaged waveform by film clip category in Cz electrode site.

The mixed model ANOVA showed a significant difference in P3 between film clip categories (F(4,112) = 15.064, p<.001) but no differences between genders (F(1,28) = .007, p = .936). The multiple pairwise post hoc comparisons showed that the P3 amplitude decreased significantly with the erotic film clips (M = 1.860, SE = .571) compared to both the social-positive (M = 2.869, SE = .584) (p = .002) and auditory alone stimuli (M = 2.947, SE = .650) (p = .038). In addition, the P3 amplitude decreased significantly with the horror film clips (M = .970, SE = .634) compared to both social-positive, social-negative (M = 2.636, SE = .668) and auditory alone stimuli (p<.001). Additionally, the P3 amplitude decreased significantly with the horror film clips compared to the erotic film clips (p = .034). Figure 1 shows the average P3 waveform amplitude in all subjects.

#### Effects of film clip categories in males on P3 amplitude at Cz

In order to prevent menstrual cycle effects, an exploratory analysis in the P3 component was performed only with the male sample. Again, the one-way repeated measures ANOVA showed a significant effect of the film clip category on the P3 amplitude (F(4,56) = 6.118, p<.001). The multiple pairwise post hoc comparisons showed a significant decrease in the P3 amplitude when participants viewed horror film clips (M = 1.162, SE = .784) compared to social-positive (M = 2.727, SE = .672) (p = .023), social-negative (M = 2.778, SE = .762) (p = .038) and auditory alone stimuli (M = 2.708, SE = .670) (p = .043). No differences in terms of P3 amplitude were found between horror and erotic film clips (M = 2.148, SE = .736) (p = .140).

#### Effects of Hemisphere×Region×Condition×Gender on P3 amplitude

**Figure 2:**
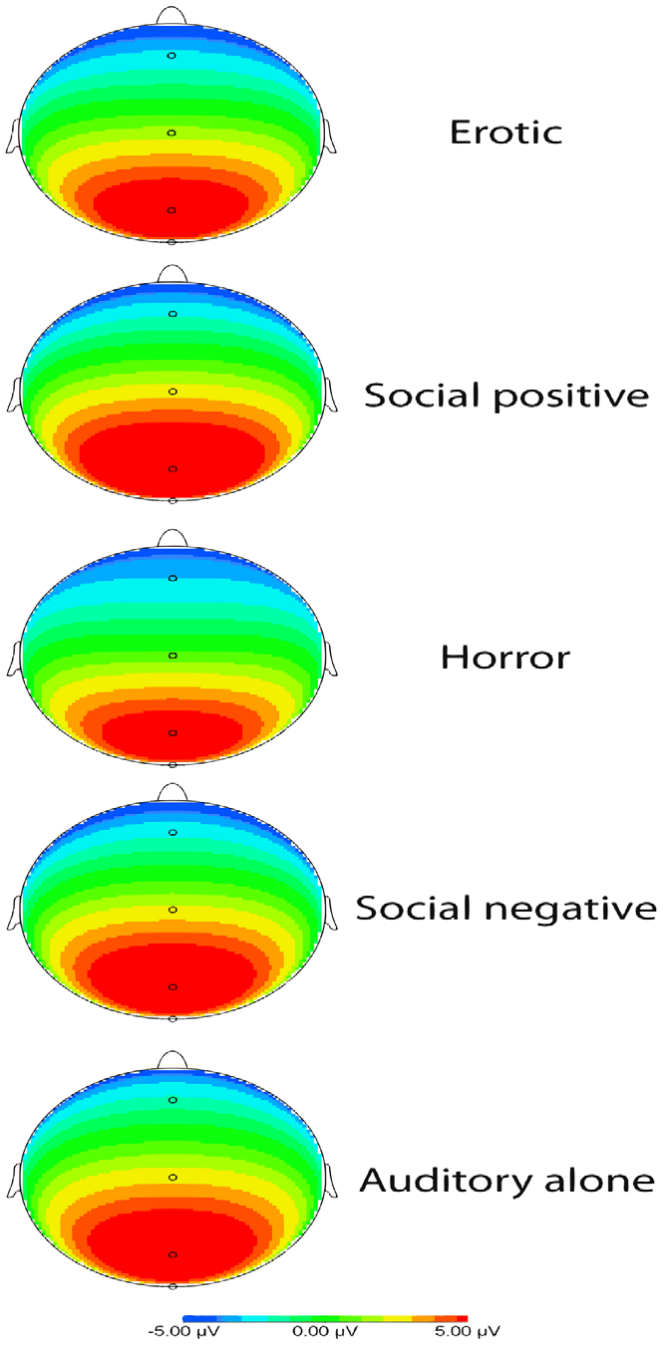
Voltage map.

In order to explore possible changes in the other regions of the scalp, an exploratory analysis with 6 regions, divided by the two hemispheres, was performed (please see the Methods section for further details). Figure 2 shows the voltage maps for each film clips category.

The mixed ANOVAs (Hemisphere×Region×Condition×Gender) did not show a significant effect of the factor Condition (F(4,112) = 2, 207, ε = .551, p = .114), neither between Hemisphere, Region and Condition (F(8,224) = 1,11, ε =  .472, p = .357). There was significant interaction between Region and Condition (F(8,224) = 2,767, ε = .455, p = .036). But the pairwise post hoc comparisons only revealed a Region effect suggesting the following gradation for all the conditions: anterior<central<posterior (p<.001). There was also a marginally significant effect for the interaction between Hemisphere and Condition (F(4,112) = 2, 458, p = .05). No significant Gender differences were found (F(1,28) = .127, p = .724).

### The Peripheral Responses

This section presents the peripheral responses elicited by the films clips while participants performed the auditory oddball task (please see the Methods section for further details).

#### Heart Rate (HR)

**Figure 3:**
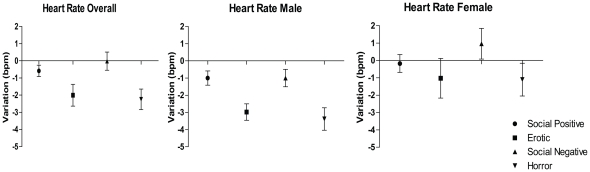
Heart rate variations by film clip category (average of 40 seconds). Means plotted with SEM error bars.

Figure 3 shows the heart rate variations for each film clip category.

The one-way repeated measures ANOVA showed a significant difference in HR between the film clips categories (F(3,84) = 9.053, ε = .682, p<.001). The multiple pairwise post hoc comparisons revealed that erotic film clips (M = −2.003, SE = .619) significantly decreased HR compared to both social-positive (M = −586, SE = .338) (p = .042) and social-negative film clips (M = −.018, SE = .509) (p = .046). In addition, horror film clips (M = −2.244, SE = .575) significantly decreased HR compared to both social-positive (p = .006) and social-negative film clips (p<.001). No differences were found between horror and erotic film clips (p = 1.000) or between social-positive and social-negative film clips (p = 1.000). A significant effect was found for the interaction between gender and HR (F(1,28) = 4.435, p = .044). In females, the post hoc comparisons revealed that horror movie clips (M = −1.103, SE = .813) significantly decrease the HR when compared to social negative clips (M = .962, SE = .720). For males, erotic film clips (M = −2.979, SE = .875) significantly decreased the HR when compared to social positive (M = −.997, SE = .464) (p = .045). Horror film clips (M = −3.384, SE = .813) significantly decreased the HR when compared to both social positive (p = .005) and negative (M = −.997, SE = .720) (p = .001). No other significant pairwise comparisons were found.

#### Skin Conductance Level (SCL)

**Figure 4:**
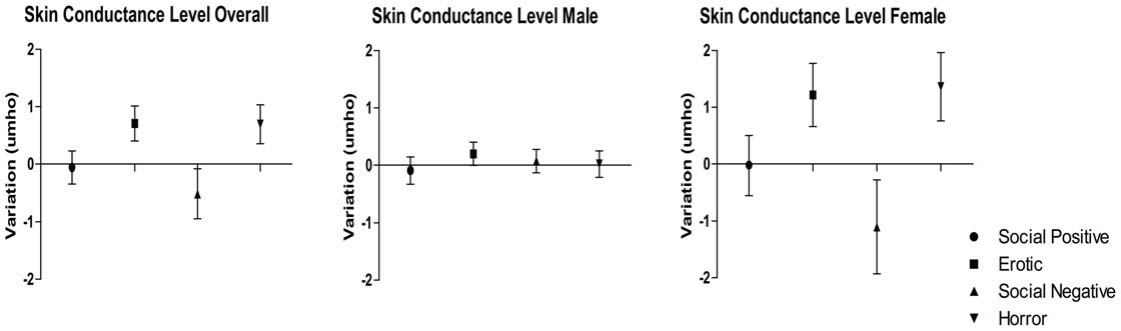
Skin conductance level variations by film clip category (average of 40 seconds). Means plotted with SEM error bars.

Figure 4 shows the SCL variations by film clips category.

The ANOVAs showed a significant difference in SCL between the film clips categories (F(3,84) = 8.974, ε = .722, p<.001). The multiple pairwise post hoc comparisons revealed that erotic film clips (M = .708, SE = .297) significantly increased the SCL compared to social-negative film clips (M = −.513, SE = .426) (p = .018),and social-positive film clips (M = −.056, SE = .288) (p = .039). In addition, horror film clips (M = .694, SE = .321) significantly increased the SCL compared to both social-positive (p = .001) and social-negative film clips (p = .004). No differences were found between horror and erotic film clips (p = 1.000) or between social-positive and social-negative film clips (p = .655). No significant gender differences were found (F(1,28) = .289, p = .595).

### Self-Reported Measures

A post film questionnaire was administered to all the participants in order to obtain the subjective scores of pleasantness and arousal for each film clip category (please see the Methods section for further details).

**Figure 5:**
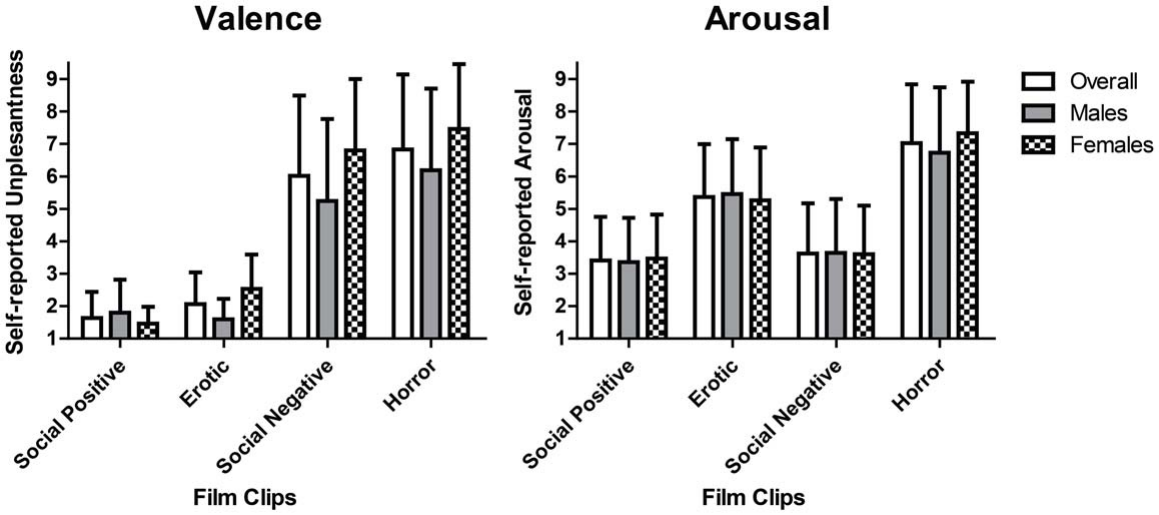
Self-report ratings of unpleasantness and arousal elicited by the film clips category. Means plotted with SEM error bars.

The Figure 5 shows the main effects of the film clips with respect to valence and arousal (F(3,87) = 63.714, ε = .681, p<.001; F(3,84) = 41.977, ε = .771, p<.001, respectively). The erotic and social-positive film clips were judged as being more pleasant than both the social-negative and horror film clips (p<.001). No significant differences were found between the social-positive and erotic film clips (p = .330) or between horror and social-negative film clips (p = 1.000). Regarding arousal, the horror film clips were rated as more arousing than the erotic, social-positive and social-negative film clips (p<.001). Additionally, erotic film clips were rated as more arousing than both social-positive and social-negative ones (p = .001); there were no differences between social-positive and social-negative film clips (p = 1.000). The film content by gender interaction was not significant for either valence (F(3,84) = .436, ε = .681, p = .677) or arousal (F(3,84) = 1.554, ε = .771, p = .220).

## Discussion

In the present study, 30 healthy, heterosexual volunteers (15 males and 15 females) watched different emotional film clips that were organized by their varying levels of pleasantness and arousal, while answering an auditory oddball paradigm (P3). The objectives of this study were to assess the P3 in a workload task, while co-registering SCL and HR measures, thus testing the potential use of this methodology as a measure of sexual motivation.

The behavioral analyses showed a high accuracy level for all the conditions with the horror condition having the largest number of errors, associated with longer RTs to the targets.

The decrease in the amplitude of the P3 component while participants viewed the different film clip categories (erotic, horror, social-positive and social-negative) is thought to represent the larger interference on cognitive processing that occurs secondary to increased workload demands [27], [28]. When taking into account the results for all the participants, the erotic film clips showed a larger interference on cognitive processing compared to both the social-positive film clip and the auditory stimulus alone (auditory oddball paradigm only). The horror film clips showed more interference on cognitive processing compared with the erotic, social-positive, and social-negative film clips as well as the auditory stimulus alone. These results are similar to those found in previous studies using emotional pictures [13], [19], [26], [29], thus supporting the concept that emotional film clips and emotional pictures have a similar effect on cognitive processing. This study did not find any differences between regions for particular conditions, thus revealing the sensitivity of the Cz electrode site for this type of workload task found in previous studies [23].

The increase in ERP responses in the setting of erotic stimuli are thought to be due to both the level of arousal [15] and pleasantness. From an evolutionary perspective, the appetitive motivational system seems to prioritize sustained attention to erotic stimuli to facilitate approach, thus promoting survival of the species [20]. Therefore, both pleasantness and the level of arousal seem to play an important role in motivational activation. However, this does not occur solely for pleasant stimuli. Previous research [30] supports the notion that it is the motivational relevance of a specific stimulus that will modulate the P3 amplitude, namely highly arousing stimuli of horror and erotic content due to their specific relevant characteristics.

Therefore, the results suggest that P3 amplitude is modulated by the motivational relevance of the stimuli and cannot be merely an index of a sex-specific behavior or sexual interest [23], [24]. The P3 component, in both erotic and horror-laden content, seems to prompt a similar “natural selective attention” [20] as long as the contents presented have similar arousal levels [31]. According to Lang et al. [20], the amount of attentional resources allocated to these stimuli is equally distributed to both appetitive and defensive brain systems during emotional processing, as both stimuli could be potentially relevant for survival. Even if sexual arousal varies in females in accordance with the menstrual cycle [32], the analysis performed on the males alone corroborated the hypothesis that P3 is not a sex-specific interest index, as there were no differences between erotic and horror film clips in males. Some studies have shown an increase in appetitive motivation in males viewing erotic content [33], but other studies have failed to replicate those results [34]. These results were however, not interpreted as sex-specific motivation but rather a gender difference in response to particular stimuli characteristics. In the present study no significant gender differences were found. The P3 amplitude reflects basic cognitive processes, such as attention, motivation and working memory, as well as changes in an organism's internal neural representation [35].

Concerning the second objective, the peripheral measures recorded in the present study revealed that the erotic and horror film clip categories produced an overall increase in the skin conductance (SCL) level and a decrease in the heart rate (HR). Overall, these peripheral measures indicate that the erotic and horror films clips used in the study effectively induced an emotional state in the participants.

However, no differences were found between erotic and horror film clips or between the social-negative and social-positive film clips. Thus, these results suggest an effect of arousal but not of valence [36] of film content.

The emotional arousal level in SCL is very sensitive to arousal and arousal has been thought to represent a strong predictor of attention and memory [34]. The HR deceleration (with an increase in electrodermal activity) is thought to represent stimulus-specific aversive responses [37], [38], [39] but is also a component of the orientating reflex [40], thus facilitating the processing of the external environment [41]. Therefore, it has been suggested that increases in SCL and decreases in HR represents increased sensory intake and attentional processing, as well as more sustained attention to the motivationally relevant stimulus [42].

Overall, when both genders were analyzed, there was a significant increase in SCL and a decrease in HR when participants watched the highly arousing film clips. It is possible that the specific gender effects found were due to the small sample size that arose when the group was split into males and females, resulting in a consequent decrease in statistical power. It is worth reminding that, contrasting with previous studies, the present research looked for emotional reactivity changes while participants were performing a workload task. This fact makes difficult to isolate entirely the effect of task from the effect of the film clip. Nonetheless, these results are partially consistent with those reported by Codispoti and colleagues [36] of an increase in SCL and decrease in HR for both genders in a passive film clip viewing task. At this point we cannot conclude that the gender differences in HR found in this study were related to the task performance. Future studies should compare the effects of film clip passive versus active task.

Regarding the self-reported measures, the general results seem to corroborate both the neurophysiological and peripheral responses. However, in the self-report, the participants did not differentiate between the pleasantness of the erotic and social-positive film clips or the unpleasantness between the horror and social-negative film clips. In the arousal ratings, the horror film clips were rated as more arousing, followed by the erotic film clips, which is consistent with both the neurophysiological and peripheral results. It is important to note, however, that previous normative ratings of these film clips has shown that horror film clips are rated as slightly more arousing than erotic ones [43]. This difference could explain the additional impact of horror movies in comparison to erotic ones. At the same time, it is difficult to find perfectly matched pleasant and unpleasant stimuli, as there are some studies showing that the sustained presentation of a negative stimulus produces a sensitization effect and increases the responses to that particular condition [44], [45]. This sensitization process may explain the greater impact of our horror film clips. Gender differences were not found in the subjective ratings in the present study. These results differ from those of previous studies [36] where females, compared to males, rated the pleasant film clips as less pleasant and the unpleasant film clips as more arousing.

The film clips in this study were not fully balanced in terms of arousal and valence, as evidenced both by the results of the SCL and the self-report measures. This limitation should be addressed in with a need for a better matching among stimuli both in terms of arousal and valence.

As shown in our study, arousal of the emotional category (erotic and horror) is a good predictor of the attentional resources allocated to the film clip, more so than sexual interest specifically. Therefore, the use of this methodology seems to be of questionable utility as a specific measure of sexual interest [23] or as an objective measure of the severity of Hypoactive Sexual Desire Disorder [24]. The present study also shows that both the SCL and HR are reactive to both sexual and non-sexual film clips and can be interpreted as reflecting similar processes of sustained attention, similar to the ones found with the P3. None of the measures used in this study revealed any kind of specific alterations that could be interpreted as sexual specific interest or motivation; thus, they should not be used in clinical practice as potential diagnostic tools.

## Methods

### Participants

A total of 30 heterosexual volunteers, 15 females (mean age: 21, 73; SD: 2, 31) and 15 males (mean age: 24, 80; SD: 3, 90), participated in the study. All participants were healthy, had normal or corrected-to-normal visual and hearing acuity, were right-handed (self-reported) and had no present or past history of neurological or psychiatric disorder. Participants had not taken any medication or psychotropic drugs during the four weeks prior to the study. Participants were also advised to avoid alcohol, cigarettes and caffeinated drinks on the day of the experiment.

### Ethics Statement

All of the participants gave their written informed consent prior to their inclusion in the study. The study was approved by the local ethics committee Centro de Investigação em Psicologia (CIPsi) and was in accordance with the Declaration of Helsinki.

### Stimulus material

#### Film clips

All participants watched fifty film clips (with a visual angle of 5°), each of which was 40 seconds in duration. The audio track from each film clip was removed in order to allow the use of an auditory oddball task. The films were divided into five categories, which are as follows: 10 erotic film clips (high arousal pleasant, with sexual intercourse but without genitalia exposure), 10 horror film clips (high arousal unpleasant), 10 social-positive film clips (i.e., people interacting with each other in pleasant ways, e.g., smiling or having fun), 10 social-negative film clips (i.e., people interacting with each other in unpleasant ways, e.g., crying or attending a funeral) and 10 blank stimuli (auditory alone). The stimuli were presented on Presentation software (Neurobehavioral Systems Inc, Albany CA).

These categories were chosen because they represent different valence categories, which would reflect the initial selective attention captured by the stimulus (namely appetitive or defensive), while the arousal level would determine the amount of attentional resources allocated to the stimulus. The unpleasant stimuli were used to allow the possibility of a distinction between approach and withdrawal responses.

### Procedure and experimental design

The participants remained seated in a comfortable chair at a distance of 1.5 m (4.92 feet) from the 48.3 cm (19 inch) screen in an electrically isolated laboratory with attenuated light and sound. They were instructed not to move during recording.

Prior to the experiment, all participants were instructed that film clips of different emotional content would be presented on a computer screen. They were instructed to fixate their eyes on the central point of the screen (marked by a cross) and that each film clip should be viewed for the entire presentation time. They were also instructed about the auditory paradigm, which consisted of two tones that were 1000 and 2000 Hz frequency, respectively. A total of 1750 auditory stimuli were presented, with a ratio of nine 1000 Hz tones for each 2000 Hz tone (35 target stimuli per category). All the stimuli were presented at the same intensity level. The interstimulus interval was 1200 msec, and each tone lasted for 100 msec.

During the training phase, all participants learned how to distinguish between the two different tones and to respond to only one of them (the 2000 Hz pure tone) by pressing the left button of the computer mouse using their dominant hand (i.e., the right hand). In the task phase, the presentation of the film clips was done in ten blocks of five film clips each, in a pseudo-randomized way: in each block, all five different categories were presented. The central cross was presented alone for 6000 msec between films clips, with a longer rest (i.e., 1 minute) between blocks. The auditory stimuli were presented during the film clips presentation after the experiment, all participants responded to a post-film questionnaire [23] concerning each film clip category. It consisted of six questions, namely about their sense of interest, arousal, enthusiasm, novelty, boredom and unpleasantness concerning the film clips. The questions were designed to examine the consistency of answers for each participant, i.e., a subject that responded highly on interest and boredom would be making an inconsistent response and, in that case, would be excluded from the study. Additionally, the ratings were used as an index of the degree of arousal and the degree of unpleasantness of each film clip.

### The Film's Valence and Arousal Ratings

All film clips were chosen with the normative ratings of arousal and valence obtained by the Self-Assessment Manikin (SAM) [46] on 113 participants on a prior normative rating of the film clips. None of those participants was included in this study. The procedure for rating the film clips was similar to the International Affective Picture System (IAPS) instructions [47], with an exposure of 40 sec, in order to match the duration of the film clips. The overall levels of valence and arousal for the film categories are presented in Table 1.

**Table 1:** Valence and Arousal mean normative ratings of the clip films categories by the Self-Assessment Manikin (SAM).

Film Clips	Mean ± SD	
	Valence	Arousal
Unpleasant High Arousal (Horror content)	2,01±1,14	7,00±1,45
Pleasant High Arousal (Erotic content)	6,53±1,10	5,73±1,45
Social Positive	6,62±0,86	3,43±1,25
Social Negative	3,04±0,81	3,97±1,21

### Physiological recording and data reduction

Electroencephalographic activity was recorded with a QuickAmp™ system with an Acticap™ System. The EEG signal was recorded at several electrode sites (Fp1, Fp2, F3, F4, F7, F8, FC1,FC2, FC5, FC6, C3, Cz, C4, CP1, CP2, CP5, CP6, T7, T8, TP9, TP10, P3, P4, P7, P8, PO9, PO10, O1 and O2) inserted in a cap with a frontopolar ground and average referenced—in accordance with the International 10–20 System [48], and passed through a 0.1–70 Hz (12 dB/octave slope) analog bandpass filter with a notch filter at 50 Hz before being sampled at 500 Hz.

Simultaneously, an ocular movement (EOG) recording was obtained with two EEG electrodes located supra- and infraorbitally to the right eye (VEOG). All impedances were maintained below 10 KΩ with high impedance active electrodes.

After signal storage, ocular artifacts were corrected offline using the Gratton and colleagues [49] algorithm. The EEG was then segmented, and 1100 ms (200 ms prestimulus baseline) epochs associated with the target stimuli (see Procedure) were extracted. Epochs with signals exceeding ±100 µV were automatically rejected, and the remaining epochs were inspected to identify and reject those still showing artifacts. The signal was passed through a 0.1–30 Hz digital band-pass filter and then corrected to the mean voltage of the pre-stimulus interval before averaging.

Only the correct trials were included in the averages, and the mean number of epochs included for each condition (after rejecting errors and epochs containing artifacts) was the following: Social-positive: 35; Erotic: 35; Social-negative: 35; Horror: 34; and Auditory Alone: 33.

The skin conductance level (SCL) and the heart rate (HR) were collected on a BIOPAC MP150 system with the AcqKnowledge 4.0 software (BIOPAC Systems Inc., California). The SCL was assessed using two Ag-Ag-Cl electrodes attached to a conductance module. The electrodes were attached to the second and third finger of the left hand, between the first and second phalanges. SCL was analyzed offline using AcqKnowledge 4.0 software (BIOPAC Systems Inc., California) and was recorded as the mean value of the entire 40 seconds of exposure to the film clip. HR was assessed using a 3-lead ECG with a lead II configuration. The average beats per minute were acquired for the entire 40 seconds of film clip exposure.

### Self-reported-measure

Following the experiment, all participants answered a self-reported questionnaire to rate the valence and arousal levels for each group of film clips. The participants rated the unpleasantness and the arousal elicited by the stimulus on two different visual analog scales, which ranged from 1 to 9 (1 being the lowest score, and 9 the highest score).

### Data analysis

After inspection of the grand averages, the mean amplitude in the 300–400 ms interval was measured, corresponding to the P3 interval. This was done because, mean amplitudes seem to be less sensitive to high-frequency noise and are unbiased for long measurement windows [50]. The mean amplitude was also measured in 6 regions of interest: left anterior (LA; Mean (F7, F3, FC5, FC1)), right anterior (RA; Mean (F4, F8, FC2, FC6)), left central (LC; Mean (T7, C3, TP9, CP5, CP1)), right central (RC, Mean (T8, C4, TP10, CP6, CP2)), left posterior (LP; Mean (P7, P3, PO9, O1)) and right posterior (RP; Mean (P8, P4, PO10, O2)).

Mixed model ANOVAs were performed with five levels (Social-positive, Erotic, Social-negative, Horror and Auditory Alone) for the Cz electrode site as a within subject factor, with two gender levels as between factors. An additional exploratory analysis with males only was performed for the P3 component, thus minimizing possible confounders in sexual arousal associated with the menstrual cycle (e.g., [32], [51]).

For the regional analysis, mixed model ANOVAs were performed with three within-subjects factors: Condition (with five levels: Social-positive, Erotic, Social-negative, Horror and Auditory Alone), Hemisphere (with two levels: right and left) and Region (with three levels: Frontal, Central and Posterior) as well as gender (female or male) as between subjects factor.

For both SCL and HR, the results are presented as a variation from the auditory alone; thus, two distinct mixed model ANOVAs (one for each measure) were performed with four within subject levels (social-positive, erotic, social-negative, horror) and with gender as between subject factor. The analysis for the self-report questionnaire was performed using two separate ANOVAs (one for arousal and the other for unpleasantness), each with one within subject factor with four levels (social-positive, erotic, social-negative, horror) and one between subjects factor with two levels (Male and Females). Two separate ANOVAS were used for the behavioral responses analysis (one for the percentage of errors, and the other for the reaction time) each with one within subject factor with five levels (social-positive, erotic, social-negative, horror and auditory alone) and one between subjects factor with two levels (Male and Females).

The Greenhouse-Geisser correction was applied to degrees of freedom in all cases in which the condition of sphericity was not met. When the ANOVAs revealed significant effects due to the factors and their interactions, posterior comparisons (i.e. post hoc) of the mean values were carried out by paired multiple comparisons (adjusted to Bonferroni). The level for statistical significance was set at p<.05, and all analyses were performed using IBM SPSS 19.0.1 (IBM®).
