# Correction: Psychophysiological Correlates of Sexually and Non-Sexually Motivated Attention to Film Clips in a Workload Task

**DOI:** 10.1371/annotation/7fba19d2-a461-41bd-b9cb-f635089aecfd

**Published:** 2012-01-18

**Authors:** Sandra Carvalho, Jorge Leite, Santiago Galdo-Álvarez, Óscar F. Gonçalves

The first two authors, Sandra Carvalho and Jorge Leite, should be noted as contributing equally to this work.

